# Supplementary material for: Towards All-Non-Vacuum-Processed Photovoltaic Systems: A Water-Based Screen-Printed Cu(In,Ga)Se2 Photoabsorber with a 6.6% Efficiency
Source: Nanomaterials (Basel). 2023 Jun 23;13(13):1920. doi: 10.3390/nano13131920 (PMC10343602; doi:10.3390/nano13131920)
Supplement: Supplementary file 1 [file nanomaterials-13-01920-s001.zip › nanomaterials-2446053-supplementary.pdf]

nanomaterials

MDPI

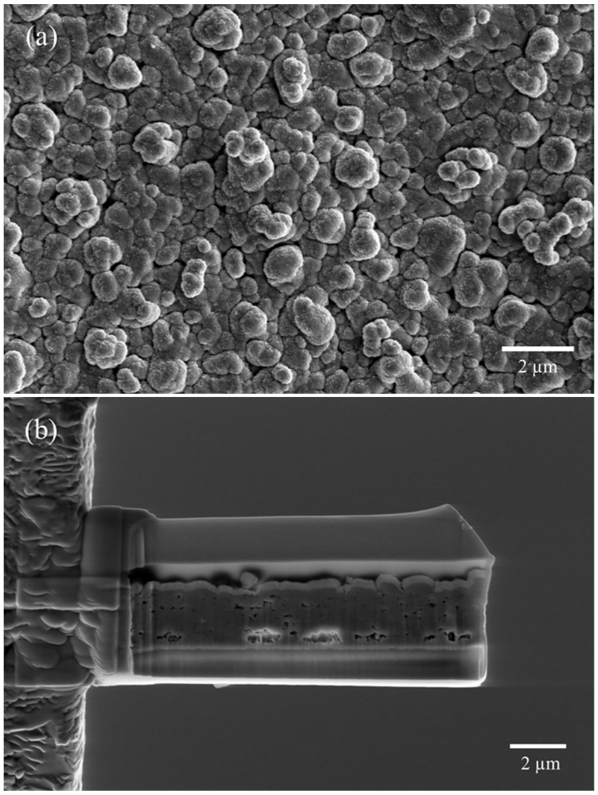

Figure S1. SEM images of SLG/FTO/CIGS/CdS/i-ZnO/AZO PV cell: (**a**) top surface and (**b**) FIB lamella preparation for cross-sectional imaging.

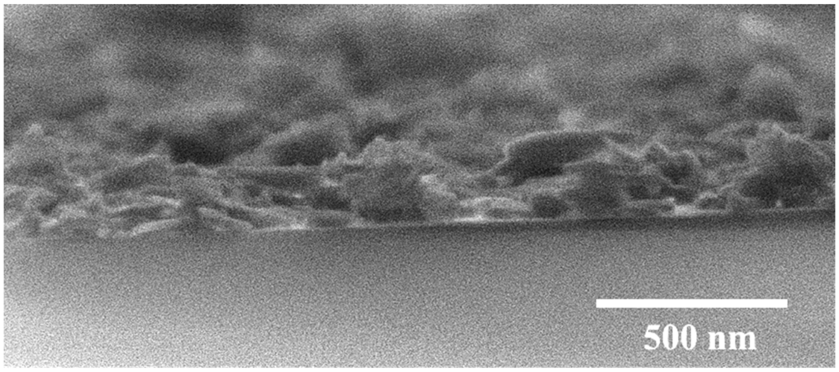

Figure S2. Cross-sectional image of spray-coated i-ZnO layer.

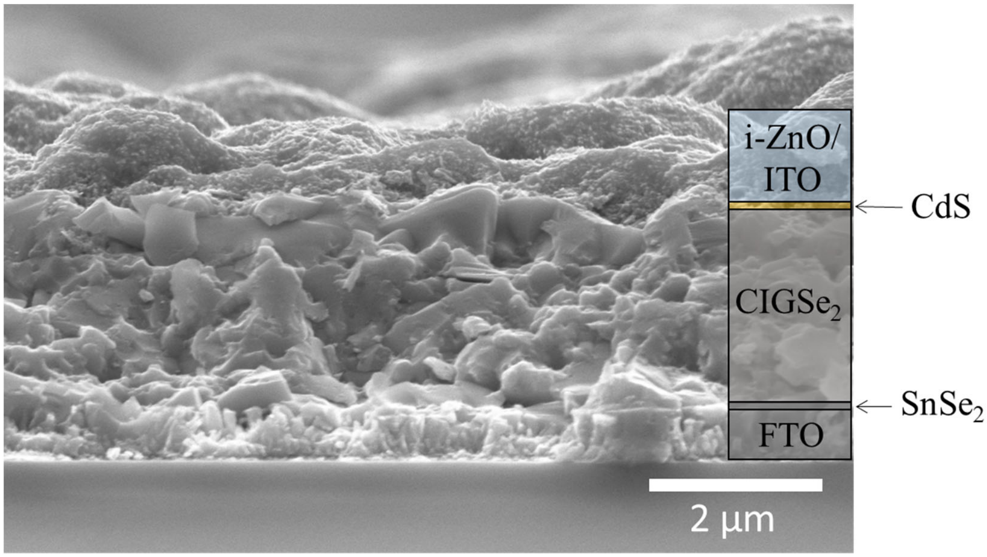

Figure S3. Cross-sectional SEM imaging of the all-non-vacuum processed CIGS PV cell.

Table S1. Photovoltaic parameters of PV cells fabricated from screen-printed photoabsorber layer and sputtered i-ZnO and AZO layers.

PV cell	Efficiency (%)	FF (%)	J_{SC} (mA cm^{-2})	V_{OC} (V)
**1**	4.9	39.2	36.0	0.33
**2**	3.6	52.1	18.4	0.36
**3**	3.6	50.8	19.7	0.34
**4**	6.6	50.2	36.7	0.34
**5**	4.3	49.5	24.0	0.35
**6**	4.5	44.5	28.6	0.34
**7**	4.8	37.8	36.5	0.33
**8**	3.8	39.2	28.5	0.33
**9**	4.1	34.0	33.8	0.34
**10**	4.6	35.3	36.6	0.34
**Average**	4.5	43.3	29.9	0.34
**Standard Deviation**	0.9	7.0	7.2	0.01

Table S2. Photovoltaic parameters of the PV cells fabricated by screen-printing of photoabsorber layer, spray-coated i-ZnO, and sputtered ITO layer.

PV cell	Efficiency (%)	FF (%)	J_{SC} (mA cm^{-2})	V_{OC} (V)
**1**	5.6	69.0	20.3	0.39
**2**	4.2	62.3	17.5	0.38
**3**	5.3	56.7	24.5	0.37
**4**	5.5	57.2	23.9	0.39
**5**	4.1	61.9	16.7	0.39
**6**	4.5	54.6	20.6	0.39
**7**	4.3	52.3	20.4	0.39
**8**	4.5	56.5	20.3	0.38
**9**	4.3	50.9	20.6	0.40
**10**	4.5	55.0	20.0	0.39
**Average**	4.7	57.6	20.5	0.39
**Standard Deviation**	0.6	5.4	2.4	0.01

Table S3. Photovoltaic parameters of PV cells fabricated by screen-printing of photoabsorber layer, and spray-coated i-ZnO and ITO layers.

PV cell	Efficiency (%)	FF (%)	J_{SC} (mA cm^{-2})	V_{OC} (V)
**1**	1.6	71.9	6.6	0.34
**2**	1.5	78.1	5.2	0.36
**3**	1.4	76.8	5.2	0.33
**4**	1.4	77.8	5.0	0.36
**5**	1.6	79.9	5.3	0.36
**6**	1.7	70.6	7.2	0.32
**7**	1.5	72.5	6.6	0.31
**8**	1.7	71.4	7.4	0.31
**9**	1.8	73.8	7.6	0.32
**10**	2.2	67.5	9.4	0.34
**Average**	1.6	74.0	6.6	0.34
**Standard Deviation**	0.2	4.0	1.4	0.02
